# Evidence that cultural groups differ in their abilities to detect fake accents: a follow up

**DOI:** 10.1017/ehs.2025.10007

**Published:** 2025-07-01

**Authors:** Jonathan R. Goodman, Robert A. Foley

**Affiliations:** 1 Department of Psychiatry, University of Cambridge, Cambridge, UK; 2 Wellcome Sanger Institute, Hinxton, UK; 3 Leverhulme Centre for Human Evolutionary Studies, University of Cambridge, Cambridge, UK

**Keywords:** signalling theory, accents, language, mimicry

## Abstract

We recently reported that cultural group membership may be a predictor of the likelihood that an individual will detect a faked accent in a recording. Here, we present follow-up data to our original study using a larger data set comprised of responses from the across the world. Our findings are in line with our previous work and suggest that native listeners perform better at this task than do non-native listeners overall, although with some between-group variation. We discuss our findings within the context of signals of trustworthiness and suggest future avenues of research.

## Graphical abstract

**Figure:**
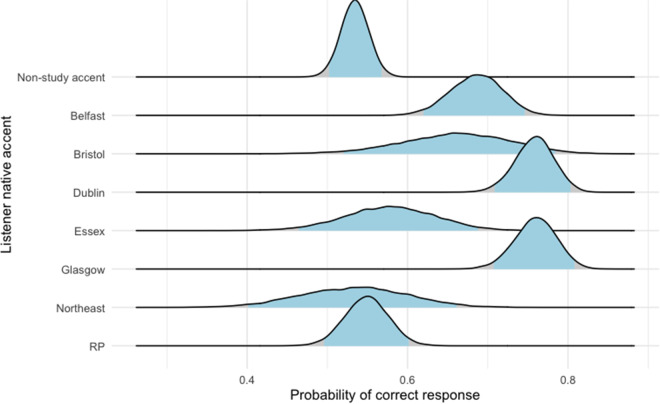

## Social media summary

Native listeners from Belfast, Dublin, and Glasgow excel at detecting fake accents, unlike RP listeners and non-natives.

We recently reported (Goodman et al., 2024) that cultural group membership may be a predictor of the likelihood that an individual will detect a faked accent in a recording. Native listeners across regions proved strong at recognising attempts at mimicking their own accents.

However, this finding was not true across all listener groups: of the Belfast, Bristol, Dublin, Essex, Glasgow, Northeast England, and Received Pronunciation (RP) accents, only speakers from Glasgow, Belfast, Dublin, and Northeast England were better at the task than were those who were non-native speakers. Notably, RP listeners of RP recordings were only slightly better than chance at detecting fakers (95% credible interval: 51%–67%), similar to non-native speakers; listeners from Belfast, Dublin, Glasgow, and Northeast England had 95% credible intervals of a correct answer ranging from approximately 62% to 85%.

Following publication of this research and extensive media engagement, we duplicated the original study via newspaper websites. The response was considerable and received approval from the University of Cambridge’s Department of Archaeology Ethics Committee for analysis of data collected (approval received in February 2025); here we present a new analysis based on 28,100 responses from 2033 participants.

We reported the study design elsewhere (Goodman et al., 2024). Our design here was identical except that we allowed participation from anywhere in the world, whereas for our previous experiment a criterion for inclusion was being raised in the UK or Ireland. Of 3020 individuals who expressed online interest in participation, 2033 answered at least one question from the experiment. The vast majority (1852) did not speak in an accent we investigated originally, with varying numbers for the seven accents evaluated (Belfast, 28; Bristol, 4; Dublin, 42; Essex, 8; Glasgow, 34; Northeast England, 5; RP, 60; see the online supplementary material for details: https://github.com/jonathanrgoodman/accents3).

First, we calculated the 95% Jeffreys interval on the entire data set, which indicated a likelihood of a correct answer of between 53.83% and 55.25%, which was lower than the interval in our previously published sample (60.32%–62.47%). We next fitted a Bayesian hierarchical model using the brms package in R (R Core Team, 2025; Bürkner et al., 2022) and the tidyr (Wickham & Girlich, 2022), ggplot2 (Wickham, 2016), and ggridges (Wilke, 2021) packages. The findings suggested that the lower probability interval for the overall sample was driven by the inclusion of the large number of participants recruited who did not speak in a study accent: in this subgroup, the 95% credible interval was 50.47%–57.25%, which was lower than among listeners who spoke in a study accent (63.99%–70.51%; difference: −15.38% to −10.64%).

A subsequent model using listener–accent group as a predictive variable showed, similar to our initial findings, that participants from Belfast, Dublin, and Glasgow drove this effect (the sample size from Northeast England was, however, too small to determine any effect). RP listeners, as with our initial findings, did not perform at a rate higher than that seen with non-study accent listeners (Table 1); these inferences are based on intervals excluding zero).

**Table 1.** 95% credible intervals for a correct answer by listener subgroup with differences, using non-study accent as a reference. Belfast, Dublin, and Glasgow native listeners all performed better at the task than did those who did not speak in a study accent

Accent	Low	High	Difference (Low)	Difference (High)
Non-study accent	0.5012296	0.5675649	Reference	Reference
Belfast	0.6195879	0.7461890	−0.20729882	−0.09244978
Bristol	0.5196256	0.7840655	−0.24827904	0.01061978
Dublin	0.7084016	0.8034444	−0.26549707	−0.18013274
Essex	0.4641964	0.6871454	−0.14832991	0.06706515
Glasgow	0.7070648	0.8077770	−0.27090651	−0.17790069
Northeast	0.4008112	0.6603625	−0.12241740	0.12907146
RP	0.4953919	0.6018196	−0.05693043	0.02888596

Finally, we obtained participant locations using longitude and latitude data collected during the online survey (however, these data points have been removed on the study’s Github page to protect participant anonymity). Of 1898 participants with location data available, 1500 were based in the United States, followed by 126 in the UK and 64 in Canada (full country-level data are available in the online supplementary material). We used this information as a proxy to group participants into one of four groups: study accent listener (169), other UK-/Ireland-based (80), other English-speaking country-based (1533), non-English-speaking country-based (116).

**Figure 1.**
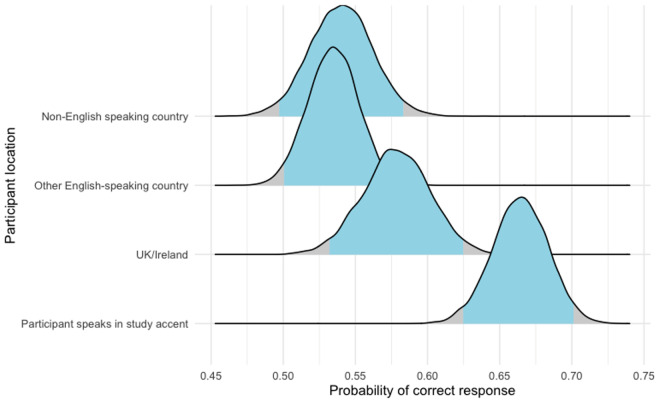
95% credible intervals for a correct response by listener subgroup. Study accent listeners had the highest probability of a correct response (62.49%–70.12%), followed by UK/Ireland (53.18%–62.44%), and other English-speaking country (50.03%–56.85%) and non-English-speaking country (49.72%–58.32%).

Using these subgroups, we fitted a final Bayesian hierarchical model using listener group as a predictive variable; the 95% credible intervals are displayed in Figure 1 and the online supplementary material. As predicted by our original findings, study accent listeners had the highest probability of success, followed by other listeners from UK or Ireland; there was, however, no notable difference between other listeners regardless of whether they were from an English-speaking country. Given that we do not know whether these listeners were from an English-speaking country originally, we cannot comment further on this finding, although we suggest this as a future avenue of inquiry.

These follow-up findings support those from our original sample, and suggest further that local importance placed on accents in cities such as Belfast, Dublin, and Glasgow, owing to cultural and historical factors, affects the likelihood that a native listener will detect accent fakery. Furthermore, the lack of difference between RP speaker–listeners and the large cohort of participants from the USA suggests that there is nothing peculiar about the Belfast, Dublin, and Glasgow accents that makes them more difficult to fake – but rather that psychological qualities among the listeners, stemming from local cultural evolution, are driving the effects noted here.

We propose that these findings justify a broader study of what common signals determine whether a signaller appears trustworthy to receivers, following suggestions that signal theory plays a major role in social selection frameworks (Gambetta & Hamill, 2005; Goodman & Milne, 2024). Accents are likely to be important signals in the formation of human bonds, helping listeners to determine the social identity of the speaker and place cooperative trust accordingly (Cohen, 2012; Pietraszewski & Schwartz, 2014a, 2014b). Other signals, including body language, use of Duchenne smiles, tattoos, and dress are, however, also likely to play a role – suggesting a broader investigation of these qualities together would be helpful in determining how humans create lasting bonds based on limited information about a partner’s trustworthiness.

## Research Transparency and Reproducibility

All data and code are available at https://github.com/jonathanrgoodman/accents3.
